# Endocrine therapy: defining the path of least resistance

**DOI:** 10.1186/bcr3659

**Published:** 2014-05-22

**Authors:** Andrew Stone, Elizabeth A Musgrove

**Affiliations:** 1 Cancer Epigenetics Program, Garvan Institute of Medical Research, 384 Victoria Street, Darlinghurst, Sydney 2010 NSW, Australia; 2 Wolfson Wohl Cancer Research Centre, University of Glasgow, Garscube Estate, Switchback Road, Glasgow, Bearsden G61 1QH, Scotland

## Abstract

One of the best-characterized oncogenic mechanisms in breast cancer is the aberrant activation of phosphatidylinositol-3-kinase, protein kinase B, and mammalian target of rapamycin signaling. In both endocrine-resistant disease and breast cancer stem cells, this is commonly caused by specific genetic lesions or amplification of key pathway components or both. These observations have generated two interesting hypotheses. Firstly, do these genetic anomalies provide clinically significant biomarkers predictive of endocrine resistance? Secondly, do tamoxifen-resistant breast cancer cells emerge from a stem-like cell population? New studies, published in *Breast Cancer Research*, raise the possibility that these hypotheses are intrinsically linked.

## 

In experimental models of estrogen receptor (ER)-positive breast cancer, the hyperactivation of phosphatidylinositol-3-kinase (PI3K), protein kinase B (AKT), and mammalian target of rapamycin (mTOR) can impede the inhibitory effect of endocrine therapy. This hyperactivation can be caused by augmented growth factor signaling, inactivation of PI3K inhibitors such as phosphatase and tensin homologue, and mutation and/or amplification of PI3K subunits and activators. Although these aberrations are frequently observed in human breast cancer, their clinical significance in endocrine-resistant disease remains controversial. Recent studies by Beelen and colleagues [1,2] and Lin and colleagues [3] in *Breast Cancer Research* raise the possibility that heterogeneity between different breast cancer cell populations may contribute to divergent findings.

Breast cancer stem cells (BCSCs) commonly harbor gain-of-function mutations in key components of the PI3K/AKT/mTOR signaling pathway [4,5]. For example, mutations in *PIK3CA*, a gene encoding a catalytic subunit of PI3K, were recently reported to be present in 8 out of 11 BCSC populations extracted from breast cancer specimens but absent in normal mammary stem cells [4]. It is thought that such attributes facilitate BCSC characteristics such as drug resistance and tumor progression [5]. A growing body of evidence suggests that endocrine-resistant breast cancer cells may be derived from a stem-like cell population [3,6]. By interrogating the genetic and epigenetic profiles of multiple tamoxifen-resistant cell lines, Lin and colleagues [3] determined that both intrinsic and acquired resistance were strongly associated with the deregulation of genes related to pluripotency and differentiation, including sex-determining region Y-box, E2F transcription factor, and retinoblastoma gene product family pocket proteins. This suggests that resistant cells could emerge from discrete populations of stem-like breast cancer cells. Because as few as 100 BCSCs were able to form tumors in mice, whereas tens of thousands of cells with alternative phenotypes failed to form tumors [7], it is apparent that endocrine-resistant BCSCs may represent a very small needle in a very large haystack. It is therefore questionable whether molecular biomarkers associated with this cell phenotype could be readily detectable in primary disease for diagnostic purposes.

Much controversy surrounds the clinical significance of PI3K/AKT/mTOR-associated biomarkers in breast cancer. Some reports, including that of Beelen and colleagues [2], suggest that *PIK3CA* mutation is associated with a longer disease-free survival, lower tumor grade, and increased ER expression [2,8,9], whereas others report an association with poor survival rates [10] (recently reviewed in [11]). Importantly, unlike other investigators, Beelen and colleagues [2] were able to examine whether these mutations were specifically associated with response to tamoxifen but did not observe a statistically significant interaction between tamoxifen treatment and *PI3KA* mutation. It would be interesting, based on the results of Lin and colleagues [3], to determine whether the same was true if *PI3KA* mutation rates were measured in BCSCs derived from the clinical trial cohorts used by Beelen and colleagues, although this is unlikely to be practicable.

Why would a *PIK3CA* mutation be predictive in some breast cancer cell types but not others? Stem cells have a greater propensity to accumulate genetic and epigenetic modifications in order to acquire a survival advantage when under selection pressure [12]. This phenomenon is further evidenced by the demonstration that emergent ‘stem-like’ resistant populations featured methylomic modifications to gene promoter regions associated with reduced expression of genes that conferred survival [3]. With the emergence of epigenetic biomarkers across multiple aspects of breast cancer research, this highlights a potential use for DNA methylation biomarkers as predictors of tamoxifen response with prospective application in neo-adjuvant studies, as discussed previously [13].

Biomarkers downstream of PI3K/AKT/mTOR signaling may reflect pathway activation more accurately than activating mutations, since the effects of active signaling will be amplified across the entire tumor cell population through paracrine growth factor receptor pathways. In agreement with this idea, Beelen and colleagues [1] demonstrated that p-p70S6K, a phosphorylation target of mTOR, was predictive of tamoxifen resistance in post-menopausal patients with breast cancer but was associated with favorable prognosis in patients from the same clinical trial who did not receive tamoxifen. One could perhaps hypothesize that there exists a mechanism by which residual ER signaling limits the expansion of a BCSC population, similar to that previously shown to regulate neural stem cells [14]. In patients who received tamoxifen, inhibiting such regulation, or indeed the acquired genetic and epigenetic changes that are accumulated by residual breast cancer cells, could then positively select for a more aggressive cell phenotype, as previously suggested in experimental models [3,13]. Irrespective of the mechanistic basis for the predictive power of p-p70S6K, as suggested by Beelen and colleagues, p-p70S6K may provide a useful companion biomarker for the mTOR inhibitor, everolimus, which has recently been approved in Europe and the US in combination with endocrine therapy for the treatment of post-menopausal women with endocrine-resistant disease [15].

Although our understanding of the origins of endocrine-resistant breast cancer is constantly evolving, it is obvious that the mechanisms that determine resistance are diverse, complex, and dynamic. Endocrine-resistant disease still represents one of the most challenging obstacles in breast cancer treatment. The appropriate use of PI3K/AKT/mTOR signaling pathway inhibitors remains to be refined in endocrine resistance; however, clinical trials suggest that their clinical impact will be considerable. To maximize their therapeutic potential, it is critical that companion biomarkers of response be further characterized.

## Abbreviations

AKT: Protein kinase B; BCSC: Breast cancer stem cell; ER: Estrogen receptor; mTOR: mammalian target of rapamycin; PI3K: Phosphatidylinositol-3-kinase; PIK3CA: Phosphatidylinositol-4,5-bisphosphate 3-kinase, catalytic subunit alpha; p-p70S6K: Phosphorylated p70S6 kinase.

## Competing interests

The authors declare that they have no competing interests.

## References

[B1] Beelen K Opdam M Severson TM Koornstra RH Vincent AD Wesseling J Muris JJ Berns EM Vermorken JB van Diest PJ Linn SC Phosphorylated p-70S6K predicts tamoxifen resistance in postmenopausal breast cancer patients randomized between adjuvant tamoxifen versus no systemic treatment Breast Cancer Res 2014 16 R6 10.1186/bcr3598 24447434 PMC3979131

[B2] Beelen K Opdam M Severson TM Koornstra RH Vincent AD Wesseling J Muris JJ Berns EM Vermorken JB van Diest PJ Linn SC PIK3CA mutations, phosphatase and tensin homolog, human epidermal growth factor receptor 2, and insulin-like growth factor 1 receptor and adjuvant tamoxifen resistance in postmenopausal breast cancer patients Breast Cancer Res 2014 16 R13 10.1186/bcr3606 24467828 PMC3978618

[B3] Lin X Li J Yin G Zhao Q Elias D Lykkesfeldt AE Stenvang J Brünner N Wang J Yang H Bolund L Ditzel HJ Integrative analyses of gene expression and DNA methylation profiles in breast cancer cell line models of tamoxifen-resistance indicate a potential role of cells with stem-like properties Breast Cancer Res 2013 15 R119 10.1186/bcr3588 24355041 PMC4057522

[B4] Pommier SJ Hernandez A Han E Massimino K Muller P Diggs B Chamberlain E Murphy J Hansen J Naik A Vetto J Pommier RF Fresh surgical specimens yield breast stem/progenitor cells and reveal their oncogenic abnormalities Ann Surg Oncol 2012 19 527 535 10.1245/s10434-011-1892-z 21748247 PMC3264874

[B5] Donovan CA Pommier RF Schillace R O’Neill S Muller P Alabran JL Hansen JE Murphy JA Naik AM Vetto JT Pommier SJ Correlation of breast cancer axillary lymph node metastases with stem cell mutations JAMA Surg 2013 148 873 878 10.1001/jamasurg.2013.3028 23884447

[B6] O’Brien CS Farnie G Howell SJ Clarke RB Breast cancer stem cells and their role in resistance to endocrine therapy Horm Cancer 2011 2 91 103 10.1007/s12672-011-0066-6 21761332 PMC10358078

[B7] Al-Hajj M Wicha MS Benito-Hernandez A Morrison SJ Clarke MF Prospective identification of tumorigenic breast cancer cells Proc Natl Acad Sci U S A 2003 100 3983 3988 10.1073/pnas.0530291100 12629218 PMC153034

[B8] Kalinsky K Jacks LM Heguy A Patil S Drobnjak M Bhanot UK Hedvat CV Traina TA Solit D Gerald W Moynahan ME PIK3CA mutation associates with improved outcome in breast cancer Clin Cancer Res 2009 15 5049 5059 10.1158/1078-0432.CCR-09-0632 19671852

[B9] Perez-Tenorio G Alkhori L Olsson B Waltersson MA Nordenskjold B Rutqvist LE Skoog L Stal O PIK3CA mutations and PTEN loss correlate with similar prognostic factors and are not mutually exclusive in breast cancer Clin Cancer Res 2007 13 3577 3584 10.1158/1078-0432.CCR-06-1609 17575221

[B10] Li SY Rong M Grieu F Iacopetta B PIK3CA mutations in breast cancer are associated with poor outcome Breast Cancer Res Treat 2006 96 91 95 10.1007/s10549-005-9048-0 16317585

[B11] Zardavas D Phillips W Loi S PIK3CA mutations in breast cancer: reconciling findings from preclinical and clinical data Breast Cancer Res 2014 16 201 10.1186/bcr3605 25192370 PMC4054885

[B12] Dean M Fojo T Bates S Tumour stem cells and drug resistance Nat Rev Cancer 2005 5 275 284 10.1038/nrc1590 15803154

[B13] Stone A Valdes-Mora F Clark SJ Exploring and exploiting the aberrant DNA methylation profile of endocrine-resistant breast cancer Epigenomics 2013 5 595 598 10.2217/epi.13.70 24283871

[B14] Brannvall K Korhonen L Lindholm D Estrogen-receptor-dependent regulation of neural stem cell proliferation and differentiation Mol Cell Neurosci 2002 21 512 520 10.1006/mcne.2002.1194 12498791

[B15] Baselga J Campone M Piccart M Burris HA 3rd Rugo HS Sahmoud T Noguchi S Gnant M Pritchard KI Lebrun F Beck JT Ito Y Yardley D Deleu I Perez A Bachelot T Vittori L Xu Z Mukhopadhyay P Lebwohl D Hortobagyi GN Everolimus in postmenopausal hormone-receptor–positive advanced breast cancer N Engl J Med 2012 366 520 529 10.1056/NEJMoa1109653 22149876 PMC5705195

